# Seed Priming with Nanoparticles and 24-Epibrassinolide Improved Seed Germination and Enzymatic Performance of *Zea mays* L. in Salt-Stressed Soil

**DOI:** 10.3390/plants12040690

**Published:** 2023-02-04

**Authors:** Bushra Ahmed Alhammad, Awais Ahmad, Mahmoud F. Seleiman, ElKamil Tola

**Affiliations:** 1 Biology Department, College of Science and Humanity Studies, Prince Sattam Bin Abdulaziz University, Al Kharj Box 292, Riyadh 11942, Saudi Arabia; 2 Plant Production Department, College of Food and Agriculture Sciences, King Saud University, P.O. Box 2460, Riyadh 11451, Saudi Arabia; 3 Department of Crop Sciences, Faculty of Agriculture, Menoufia University, Shibin El-Kom 32514, Egypt; 4 Precision Agriculture Research Chair, Deanship of Scientific Research, King Saud University, Riyadh 11451, Saudi Arabia

**Keywords:** ZnO NPs, 24-Epibrassinolide, phytochromes, germination, metabolic activity, maize

## Abstract

Saline stress is one of the most critical abiotic stress factors that can lessen crops’ productivity. However, emerging nanotechnology, nano-fertilizers, and developing knowledge of phytochromes can potentially mitigate the negative effects of saline stress on seed germination. Therefore, the aim of this study was to investigate the effects of seed priming either with zinc oxide nanoparticles (ZnO-NPs; 50 and 100 mg L^−1^) or 24-epibrassinolide (EBL; 0.2 and 0.4 μM) and their combinations on maize (*Zea mays* L.) grains sown in salt-stressed soil (50 and 100 mM NaCl). Saline stress treatments significantly affected all germination traits and chemical analysis of seeds as well as α-amylase activity. Compared to un-primed seeds, seed priming with ZnO-NPs or EBL and their combinations significantly increased the cumulative germination percentage, germination energy, imbibition rate, increase in grain weight, K^+^ content, and α-amylase activity, and significantly reduced germination time, days to 50% emergence, Na^+^ uptake, and Na^+^/K^+^ ratio of maize sown in salt-stressed-soil (50 or 100 mM NaCl). The combination of 100 mg ZnO-NPs L^−1^ + 0.2 μM EBL resulted in the highest improvements for most of the studied traits of maize seeds sown in salt-stressed soil in comparison to all other individual and combined treatments.

## 1. Introduction

The growing human population, climate change, natural disasters, anthropogenic activities, agricultural land degradation, and salinity are posing extra pressure on food security and agricultural sustainability worldwide [1,2]. Maize (*Zea mays* L.) is ranked third after wheat (*Triticum aestivum* L.) and rice as a staple food for humankind and has become a potential vital cereal crop due to its diversified uses, wide adaptability, and low cost of production [3]. It contributes 30% of the daily calories of 4.5 billion people globally [4]. Despite important genetic developments, maize is still susceptible to environmental stresses such as salinity [5].

Soil salinity, among the abiotic stresses, is considered a major factor that can significantly affect the germination, physiology, and productivity of strategic crops [6,7,8]. At present, about 7% of the global land surface (approximately 1 billion ha) is salt-affected. Even though most of this has resulted from quite consistent and slow natural geochemical processes, nearly 30% of it was categorized as human-induced salinization, including 189 M ha in the Middle East [9,10].

Salinity stress can significantly affect soil quality and nutrition availability which consequently can affect plant performance in all growth stages such as germination, seedling establishment, vegetative growth, and yield [11,12]. This can be due to the reduction in the osmotic potential of the ambient soil water which consequently can reduce seed imbibition. Moreover, the excess Na^+^ and Cl^−^ ions absorbed with water uptake can cause ionic toxicity and thus can negatively affect the metabolic processes in germinating seeds through enzymatic activity such as respiration, hydrolysis of nutrients, and energy production [13,14]. Furthermore, salinity stress can inhibit seed germination, delay the germination time, and reduce the germination percentage and speed [15,16]. Such negative impacts of salinity stress on seed germination or plant performance can occur through the dysfunction in the physiological and biochemical processes and the antioxidant defences due to the excessive production of ROS along with the variabilities of cell membranes and lipid peroxidation that can be occurred because increased Na^+^ ions along with increased ROS [17]. Plants can have some antioxidative mechanisms for ROS scavenging by inspiring the antioxidative enzymes such as CAT, SOD, and POX, which function along with the non-enzymatic antioxidants to alleviate the negative effects of abiotic stresses in plant species [18].

Different approaches, including the conventional breeding of selective cultivars, genetic modifications using molecular techniques, use of plant-growth-promoting rhizobacteria and arbuscular mycorrhizal fungi, nutrient management, seed priming, and exogenous application of phytochromes can be used to mitigate saline stress in crop plants [1]. Therefore, seed priming with either nanoparticles or brassinosteroids (BRs) can be a potential strategy to overcome the hostile nature of salinity stress and provide a beneficial option in the form of nutrients, metabolites, and even hormones [19,20]. Seed priming can include hormones, growth regulators, nutrients, metal ions, nanoparticles, fertilizers, bacteria, and even radiation [21]. The physiological, biochemical, and molecular responses of different crop seeds to the application of seed priming made it a widely accepted approach to cope with both biotic and abiotic stress in plants [22].

Although nanoparticles, particularly nano-fertilizers, are a relatively new application in agriculture, their efficacy and effectiveness for mitigating abiotic stress have received considerable interest from the agricultural community [23]. For instance, ZnO-NPs can be used as nano-fertilizers or as mitigators for plant stress factors [24,25] This might be due to its role as an essential micronutrient for plants that can serve as a co-factor of several metabolic and regulatory enzymes and thus can be involved in a number of physiological processes from seed germination to harvest processes [23,26,27,28]. ZnO-NPs can promote seedling traits when applied at 10–200 ppm for tomato seedlings [29] or 5–20 mg L^−1^ for wheat germination and seedlings growth [30]. On the other hand, brassinosteroids (BRs) are endogenous phytohormones and essential plant hormones that can regulate multiple physiological processes at both cellular and organismic stages when plants are grown under abiotic stress [10]. The application of 24-Epibrassinolide (EBL), an active by-product of brassinolide biosynthesis, can mimic the stimulating effect of BRs in plants [31]. In this respect, Özdemir et al. [32] and Derevyanchuk et al. [33] reported that EBL can enhance seed germination, seedling growth, lipid peroxidation, proline content, and antioxidative systems of plants such as rice (*Oryza sativa* L.) or arabidopsis (*Arabidopsis thaliana* L.).

Therefore, the present study aimed to investigate the potential efficacy of seed priming either with ZnO-NPs (50 and 100 mg L^−1^) or EBL (0.2 and 0.4 μM) and their combinations on germination traits and enzymatic performance of maize grown in salt-stressed soil (50 and 100 mM NaCl).

## 2. Results

### 2.1. Increase in Grain Fresh Weight

**Figure 1:**
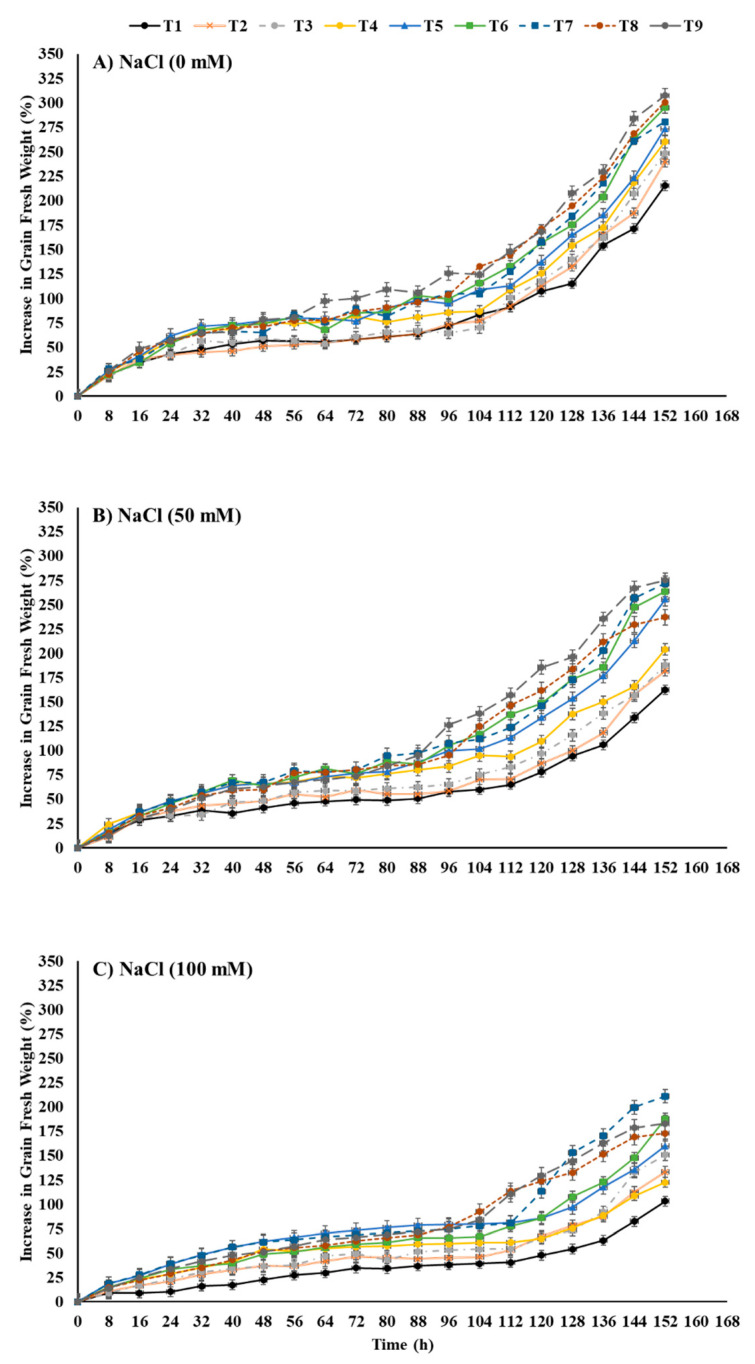
Effects of ZnO-NP and EBL seed-priming treatments on the increase in grain fresh weight of maize seeds sown under different salinity-stress conditions (**A**–**C**). T1 = Control (only distilled water), T2 = EBL (0.2 μM), T3 = EBL (0.4 μM), T4 = ZnO-NPs (50 mg/L), T5 = ZnO-NPs (100 mg/L), T6 = EBL (0.2 μM) + ZnO-NPs (50 mg/L), T7 = EBL (0.2μM) + ZnO-NPs (100 mg/L), T8 = EBL (0.4 μM) + ZnO NPs (50 mg/L), and T9 = EBL (0.4 μM) + ZnO-NPs (100 mg/L).

The increase in grain fresh weight of maize seeds depicted a percentage (*w*/*w*) increase compared to grain dry weight measured directly before sowing (Figure 1). The increase in grain fresh weight was negatively affected by salt-stress treatments. For instance, salt-stress treatments with either 50 mM or 100 mM reduced the increase in the grain fresh weight by −57.31 and −108.42%, respectively, in comparison to the control at 152 h after sowing. However, seed priming with different treatments of ZnO-NPs and EBL individually or in combination significantly enhanced the increase in the grain fresh weight of maize sown in salt-stressed soil. Seeds primed with a combination of 100 mg ZnO-NPs L^−1^ + 0.04 μM EBL (T9) showed a remarkable increase by +42.80% for the increase in the grain fresh weight in comparison to un-primed seeds sown in unstressed soil (control treatment). Moreover, the application of the combination treatment resulted in the highest increase in fresh grain weight of maize sown in soil treated with 50 mM NaCl by +69.39% in comparison to the unprimed seed treatment. However, seed priming with 100 mg ZnO-NPs L^−1^ + 0.02 μM EBL increased the increase in grain fresh weight of maize sown in the highest level of salt-stressed soil (100 mM NaCl) by +104.37% in comparison to the unprimed seed treatment.

### 2.2. Imbibition Rate

**Figure 2:**
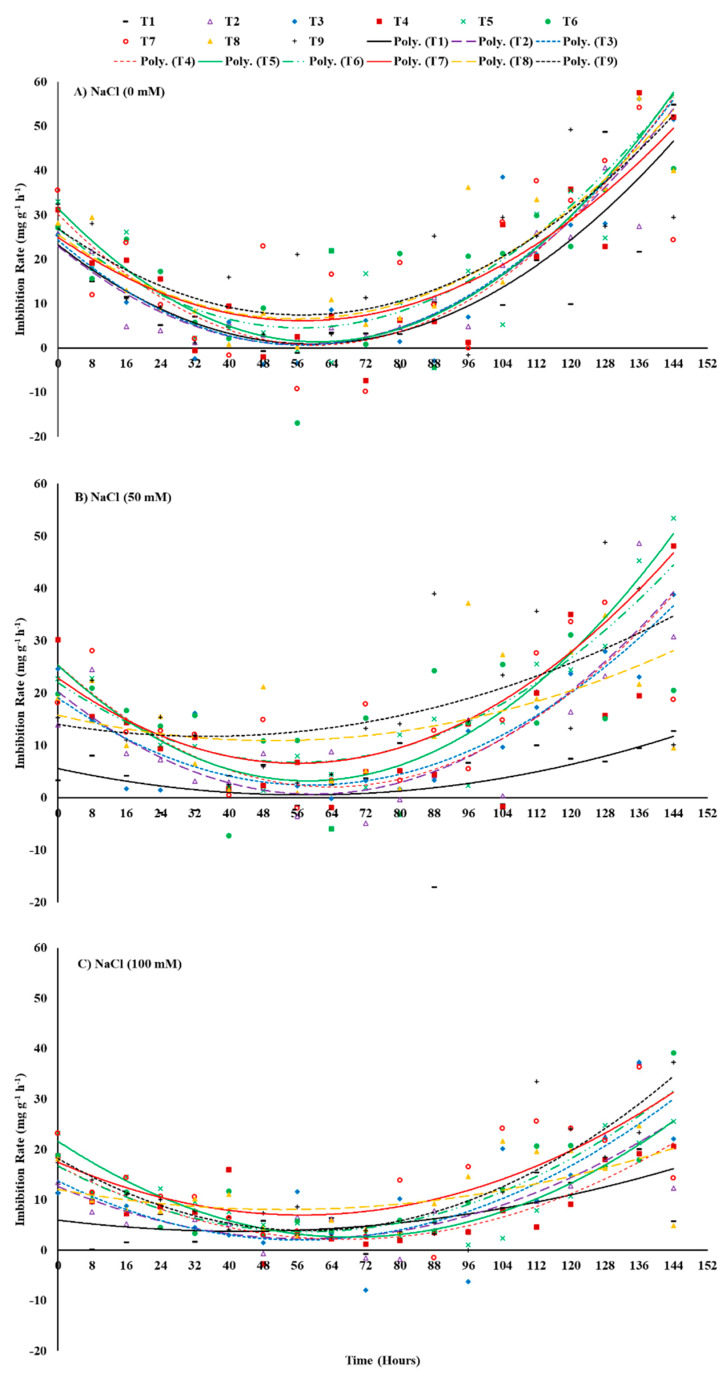
Effects of ZnO-NP and EBL seed-priming treatments on the imbibition rate of maize seeds sown under different salinity-stress conditions (**A**–**C**). The trend lines represent polynomial grade 2 relationships. For abbreviations, please see Figure 1.

Data have been presented as a scatter plot with lines of best fit representing seed-priming treatments as quadratic equations (grade 2) over the period of 144 h starting from the sowing point (Figure 2). The slope of the lines shows how the rate of imbibition varied during the process of seed germination and to what extent saline stress and seed-priming treatments modified this behavior. Under different salt-stress treatments, all primed seed treatments showed a gradual decline in imbibition rate up to 56 h which reached nearly zero on average and then increased once again. Seed priming with ZnO-NPs as individual application either at 50 or 100 mg ZnO-NPs L^−1^ significantly enhanced the imbibition rate of maize sown in salt-stressed soil with 50 mM NaCl in comparison to other individual or combined treatments of ZnO-NPs and EBL. However, seed priming with combined treatment of 100 mg ZnO-NPs L^−1^ + 0.04 μM EBL resulted in better imbibition rates for maize sown in the highest level of salt-stressed soil in comparison to other primed seed treatments (Figure 2).

### 2.3. α-Amylase Activity

**Figure 3:**
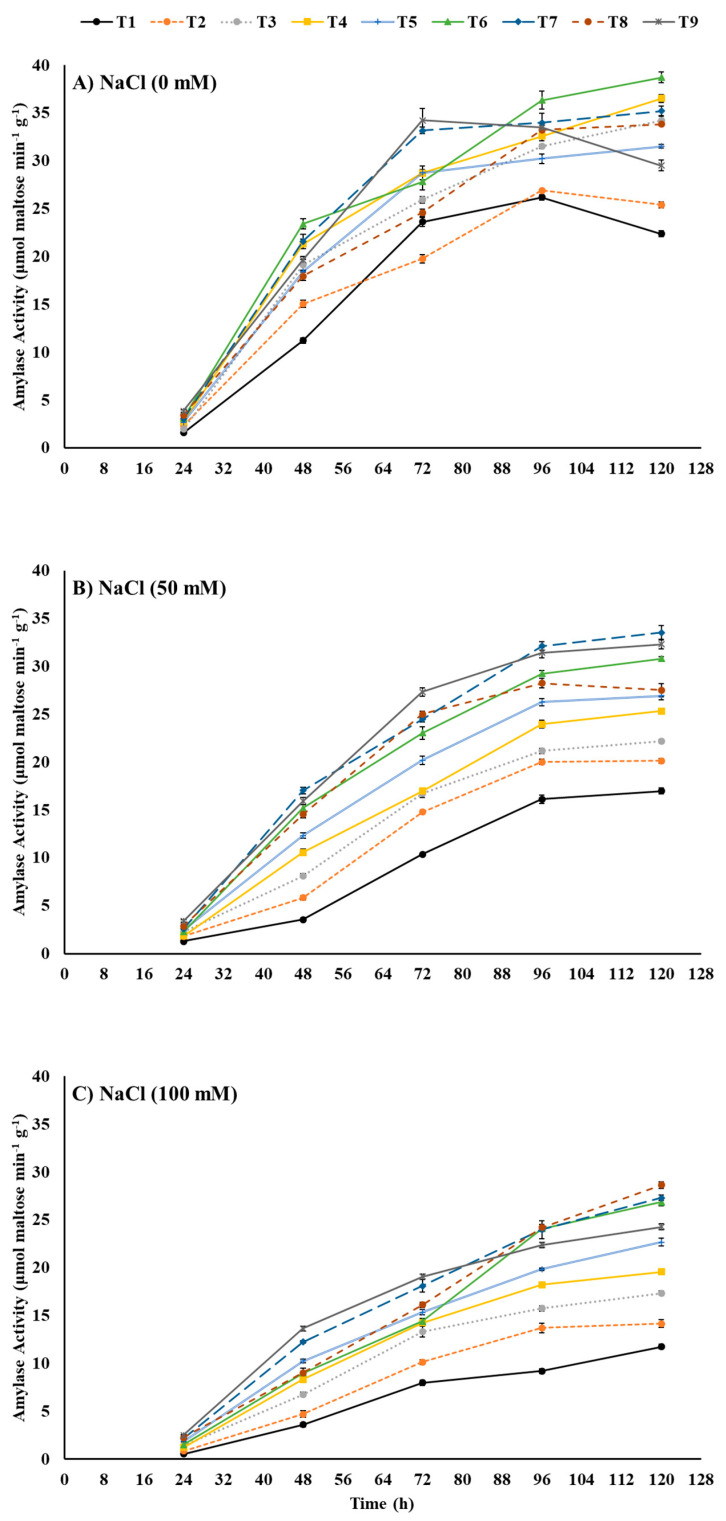
Effects of ZnO-NP and EBL seed-priming treatments on α-amylase activity of maize seeds sown under different salinity-stress conditions (**A**–**C**). Error bars represent the standard error (±SE). For abbreviations, please see Figure 1.

Alpha amylase aleurone of seeds plays an important role in the hydrolysis of endosperm starch to provide sugars for metabolism which provides energy for germinating seeds. High saline stress may slow down the amylase activity and hence the germination attributes. We choose α-amylase activity aiming to show that seed priming with ZnO-NPs, EBL, or their combination could possibly improve seed germination by mitigating the negative effect of saline stress on amylase activity. The α-amylase activity of maize seeds, considering the fundamental metabolism in germinating seeds, was tested after each 24 h for the first five days. The results portray the detrimental effects of saline stress on α-amylase activity, which escalated with the intensification of stress from 0–100 mM NaCl (Figure 3). After 120 h, 50 mM NaCl and 100 mM NaCl reduced the α-amylase activity by −24.11% and −47.77%, respectively, compared to the control (0 mM NaCl). In contrast, seed priming with ZnO-NPs, EBL, and their combinations proved themselves a mitigator of saline stress by amelioration of the saline-stress-mediated decline in α-amylase activity. Stimulatingly, seed-priming treatments elevated the α-amylase activity even under no saline stress (control) where T6 (ZnO-NPs at 50 mg L^−1^ + EBL at 0.2 μM) outperformed all other treatments and resulted in +72.77% higher activity compared to the control after 120 h. Under medium saline stress (50 mM NaCl), T7 (ZnO-NPs at 50 mg L^−1^ + EBL at 0.4 μM) followed by T9 (ZnO-NPs at 100 mg L^−1^ + EBL at 0.4 μM) enhanced the α-amylase activity by +96.41% and +90.33%, respectively, compared to the control after 120 h of sowing. Seed-priming treatment T8 (ZnO-NPs at 100 mg L^−1^ + EBL at 0.2 μM), followed by T7 (ZnO-NPs at 50 mg L^−1^ + EBL at 0.4 μM), showed remarkably higher α-amylase activity under high saline stress (100 mM NaCl) with maximal increases of +144.44% and +133.33%, respectively, compared to the control at 120 h after sowing.

### 2.4. Germination Characteristics

The salt-stress treatments negatively affected the cumulative germination percentage, days to 50% emergence, mean germination time, and germination energy of maize seeds; however, seed-primed treatments with ZnO-NPs, EBL, and their combinations significantly reduced the negative effects of salt-stress treatments (Table 1). The highest increase in cumulative germination percentage was obtained from primed seeds with T7 (+17.95%) and T9 (+17.66%) followed by T5 (+17.08%) and T6 (+16.77%) when the soil was not stressed with salinity and T9 (+16.77%) when the soil was stressed with 50 mM NaCl in comparison to un-primed seeds sown in the highest level of salt-stressed soil, control, (Table 1). In salt-stressed soil with 50 mM NaCl or unstressed soil, most of the primed-seed treatments with ZnO-NPs, EBL, and their combinations resulted in a lower number of days to 50% germination and mean germination time without significant differences among them. On the other hand, primed seeds with T3 and T8 resulted in a significant and lower number of days to 50% germination and mean germination time in comparison to primed seeds treatment with T5 or unprimed seeds when sown in 100 mM NaCl (Table 1). Interestingly, the maximum elevation in germination energy (+19.15%) was obtained from primed seeds with T8 when sown in salt-stressed soil with 50 mM NaCl in comparison to those obtained for unstressed soil and un-primed seed. In contrast, the maximum decrease (−95.75%) was recorded for maize seeds primed with T1 and T5 when sown in salt-stressed soil with 100 mM NaCl.

### 2.5. Daily Germination Performance

**Figure 4:**
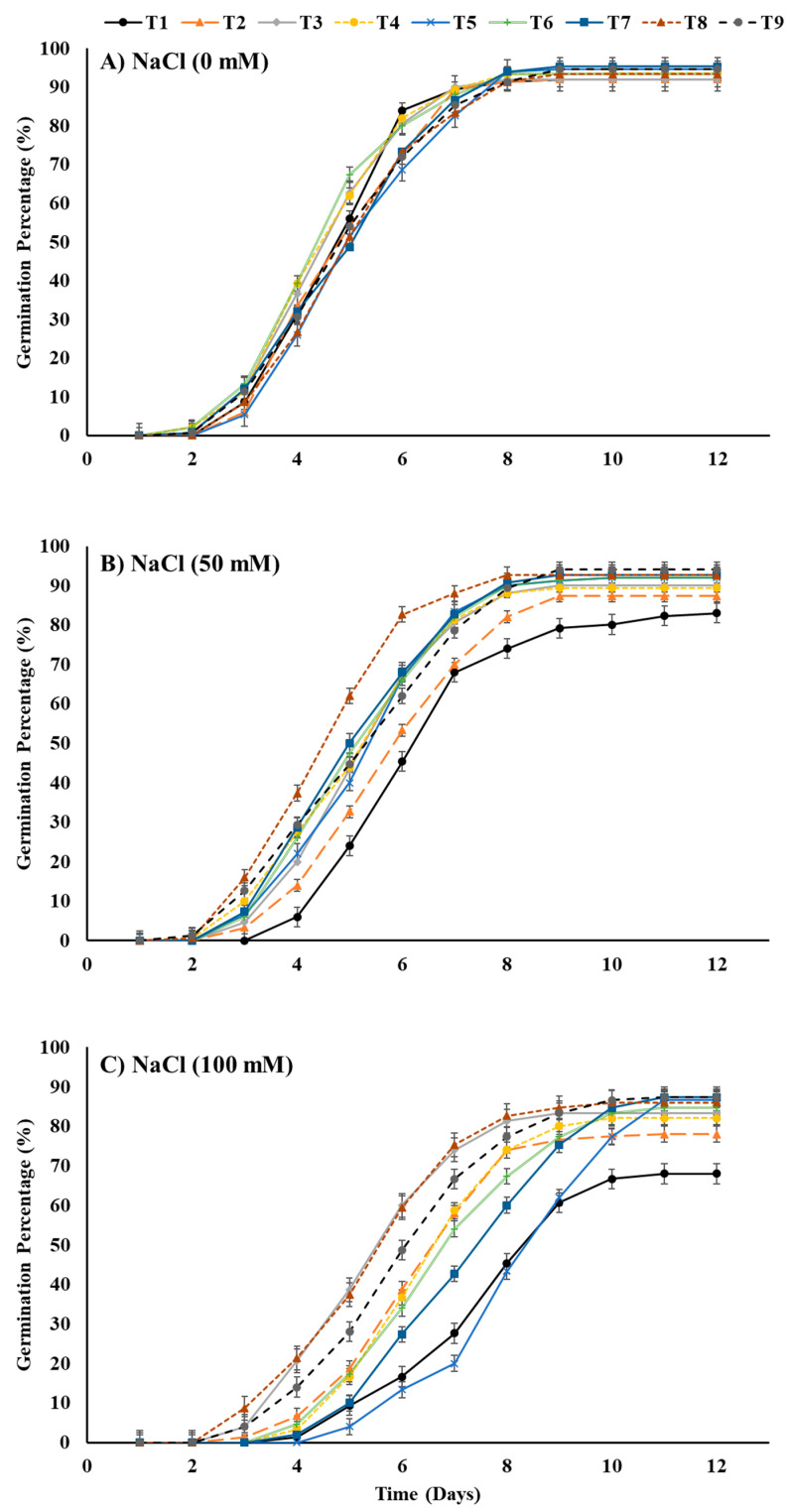
Effects of ZnO-NP and EBL seed-priming treatments on the daily germination response of maize seeds sown under different salinity-stress conditions (**A**–**C**). For abbreviations, please see Figure 1.

The results obtained showed a noteworthy delay in germination as well as a decline in the germination percentage caused by salt-stress treatments (Figure 4). However, seed priming with T8 (ZnO-NPs at 100 mg L^−1^ + EBL at 0.2 μM) and growth in salt-stressed soil with 50 mM NaCl and priming seeds with T8 or T3 (EBL at 0.4 μM) and growing them in salt-stressed soil with 100 mM NaCl resulted in a higher germination percentage compared to all other seed-priming treatments.

### 2.6. Ion Accumulation

**Figure 5:**
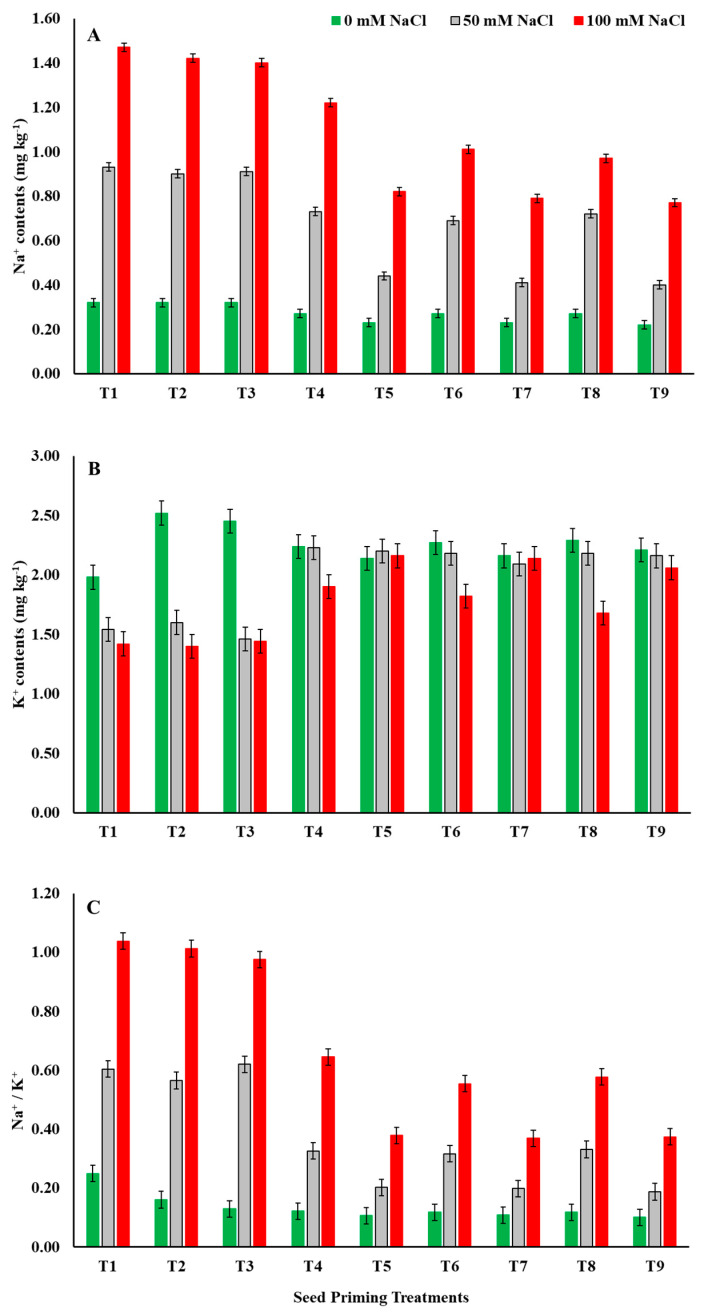
Effects of ZnO-NP and EBL seed-priming treatments on the Na^+^ (**A**) and K^+^ (**B**) contents and the Na^+^/K^+^ (**C**) of maize seeds sown under different salinity-stress conditions. Error bars represent the standard error (± SE). For abbreviations, please see Figure 1.

Salinity stress increased the Na^+^ accumulation significantly in maize seeds (Figure 5A). The highest Na^+^ contents (1.47 mg kg^−1^) were obtained from seeds sown in soil treated with 100 mM NaCl with no priming treatment. On average, medium salinity (50 mM NaCl) elevated Na^+^ concentration by +150.37% while high salinity (100 mM NaCl) resulted in 303.31% higher Na^+^ compared to the control. Seed priming with ZnO-NPs, EBL, and their combinations significantly hampered the saline-stress-dominated Na^+^ accumulation. Seed priming with a combination of ZnO-NPs at 100 mg L^−1^ + EBL at 0.4 μM (T9) resulted in −56.99% and −47.62% lower Na^+^ concentration compared to control (T1) under 50 mM NaCl and 100 mM NaCl saline stress, respectively. In general, seed-priming treatments with high ZnO-NP concentrations such as T5, T7, and T9 consistently and significantly performed better than all other individual and/or combined treatments, especially under saline-stress conditions (Figure 5A).

Unlike Na^+^ accumulation, seed K^+^ contents were significantly decreased by prevailing saline stress. Medium soil salinity (50 mM NaCl) showed −12.93% while high salinity resulted in −20.92% lower K^+^ content compared to no salinity (control). Contrarily, seed-priming treatments remarkably ameliorated the saline-stress-mediated decline in K^+^ contents of seeds and maintained higher K^+^ contents compared to un-primed seeds (T1) even under high salinity (Figure 5B). Seed priming with ZnO-NPs at 50 mg L^−1^ (T4) resulted in a maximum increase (+34.53%) in K^+^ contents in medium saline stress (50 mM NaCl) whereas the maximal depression (−5.19%) was noted for T3 (EBL at 0.4 μM). A similar outcome was also seen under high salinity (100 mM NaCl) with the maximal elevation of +35.18 by seed priming with ZnO-NPs at 100 mg L^−1^ (T5) compared to the control. Conclusively, the seed-priming treatments with high ZnO-NP concentrations (ZnO-NPs at 100 mg L^−1^) resulted in relatively larger effect in mitigating the deleterious effects of salinity compared to EBL in combination with ZnO-NPs and EBL alone (Figure 5B).

Maize seeds’ Na^+^/K^+^ were significantly affected by both saline stress and seed priming with ZnO-NPs and EBL. The results obtained showed that, as the saline stress intensity increased from 0–100 mM NaCl, the Na^+^/K^+^ became wider and wider (Figure 5C). Medium salinity (50 mM NaCl) increased the ratio by +177.61% while high saline stress (100 mM NaCl) resulted in a +391.04% wider Na^+^/K^+^ compared to the control. However, Na^+^/K^+^ was narrowed down by seed-priming treatments in all three saline intensities. Even under no saline stress, seed priming with T9 (ZnO-NPs at 100 mg L^−1^ + EBL at 0.4 μM) minimized it by −59.84% compared to the control. Following the same trend, T9 resulted in −69.04% and −64.45% narrower Na^+^/K^+^ ratios for 50 mM NaCl and 100 mM NaCl, respectively, compared to the control (T1).

## 3. Discussion

Germination is an aggregative complex of several biochemical and physiological phenomena such as the reactivation of cellular respiration, mobilization of metabolites, synthesis of mRNA and proteins, and activation of cell division [34]. Salinity is one of the major abiotic stresses that can significantly reduce agricultural crop productivity [35]. However, the deleterious effects of salt stress depend on a number of plant factors such as seed germination, growth stage, tolerance index, homeostatic response, and even genetics. Among cereal crops, maize has been used as a model plant to investigate the effect of abiotic stresses on crop plants [36]. Salinity poses osmotic stress and ionic toxicity in response to which numerous defense mechanisms activate in germinating seeds, including the upregulation of genes related to defensive proteins, hormones, and energy metabolism. The production of antioxidant enzymes, cytoplasmic detoxification, maintenance of cellular turgor and structures, and higher metabolic activity are the primary natural responses commonly shared by all plant species. Seed priming could possibly assist the germinating seed to mitigate saline stress by neutralizing ionic toxicity or by promoting defense mechanisms [37].

In the current study, the highest level of salt stress (100 mM NaCl) followed by 50 mM NaCl significantly affected all investigated traits in comparison to the control treatment (unstressed soil). Our results demonstrated that salt stress significantly reduced the increase in grain fresh weight (Figure 1) by reducing the water uptake and imbibition rate in maize compared to the control (Figure 2). Furthermore, it lowered the cumulative germination percentage and germination energy while delaying the mean germination time and days to 50% emergence (Table 1). These findings are evidently supported by lower α-amylase activity and Na^+^ accumulation in the germinating seeds under salt stress compared to the control (Figure 4 and Figure 5). This could possibly be due to the imposition of osmotic irregularities, ionic toxicity, and/or impaired metabolism in germinating seeds [36]. Salinity accumulates Na^+^ and Cl^−^ ions in the soil solution, which imposes osmotic pressure, delays imbibition, and reduces water absorption; thus, seeds dehydrate in soil that contains enough water for germination [38]. Consequently, it can reduce the germination percentage and germination speed as well as delay the germination process by reducing the necessary metabolic activities [39].

The increased Na^+^ ions inside the seed can induce ionic toxicity which can dismantle the cell membrane integrity due to the denaturation of membrane proteins and peroxidation of phospholipids [40]. Subsequently, ion homeostasis, uptake of essential nutrients, mechanism of active transportation, and osmoregulation are interrupted near completion depending on the severity of salt stress [41]. The unusual balance of various ions, such as the Na^+^/K^+^ ratio, is responsible for secondary stresses that can minimize the metabolic activities of enzymes resulting in low germination of seeds sown in salt stress [42]. A general trend of increased Na^+^ content and Na^+^/K^+^ ratio was observed in our study (Figure 5). Furthermore, an elevated pressure on homeostatic mechanisms costs an additional amount of energy and negatively affects carbohydrate hydrolysis and oxidation [15,38]. Thus, a lower α-amylase activity yields less glucose and subsequent glucose respiration resulting in a slower germination process [43]. The results presented in Figure 4 showed that salt stress reduced the α-amylase activity by up to 40% compared to those obtained from the control. Our findings are in line with Kubala et al. [35], who reported a 20% reduction in germination percentage in *Brassica napus* L. when exposed to 100 mM NaCl salt stress. On the other hand, Anaya et al. [44] reported that 100 mM NaCl caused a reduction in the germination percentage by 60.61% and mean germination time by 16.67% in *Vicia faba* L. Furthermore, El-Hendawy et al. [45] reported a decline of 41.30% in α-amylase activity after 96 h of wheat germination under 120 mM NaCl saline stress.

In addition to emulation of imbibition, seed priming poses moderate stress on seeds which triggers a stress-specific response in seeds that helps later to tolerate future stress. Prior to germination, it activates seed metabolism, RNA production, antioxidants, and protein synthesis, which consequently ensures proper seed germination and development [46]. The current study has proven a significant improvement in water absorption, higher α-amylase activity, and better germination performance of maize seeds in response to seed priming of maize with ZnO-NPs, EBL, and their combinations when sown in salt-stressed soil.

Zn is an essential micronutrient in plants, and although it is required in very low concentrations, it plays an important role in key metabolic activities and osmoregulation at the cellular level [47]. Zn, being a co-factor of biocatalytic enzymes, e.g., isomerases, transferases, ligases, and hydrolases, is vital for metabolic activities in germinating seeds [30,48]. Thus, an external application of Zn, such as seed priming, can help the seeds to develop cross-tolerance against abiotic stresses such as ionic toxicity, saline stress, and osmotic stress.

ZnO-NP priming treatments (T4, T5) showed a significant improvement in water absorption of maize seeds (Figure 1), imbibition rate (Figure 2), germination percentage, and germination energy (Table 1) compared to control. ZnO-NPs further improved the germination performance by reducing the mean germination time and regulating germination speed on a daily basis (Figure 3 and Table 1). They also enhanced the K^+^ ion uptake by reducing the Na^+^ absorption and hence regulated a lower Na^+^/K^+^ ratio even under 100 mM NaCl stress (Figure 5). Furthermore, a sufficiently higher α-amylase activity under salinity stress was noted in response to seed priming with the individual application of ZnO-NP treatments (Figure 4). In general, seed priming with ZnO-NPs at 100 mg L^−1^ resulted in better performance than ZnO-NPs at 50 mg L^−1^ and the control for most of the studied parameters. Li et al. [49] reported a significant improvement in the germination percentage (5.59%) and α-amylase activity by 30.29% in rice primed with ZnO-NPs at 100 mg L^−1^. A notable increase in germination percentage (7.23%) and germination speed (25.53%) has also been observed in *Brassica napus* under salinity (150 mM NaCl)-stress conditions at a rate of 100 mg L^−1^ ZnO-NPs [50].

The enhancement in the investigated traits as a result of the ZnO-NP application in the current study could be due to the slower release of such NPs upon seed priming and hence could last longer to establish seedlings and even during vegetative growth, as reported by [51]. Savassa et al. [51] conducted microscopic and X-ray studies to prove that seed priming with ZnO-NPs accumulated Zn in the seed coat and released it slowly but for a longer period of time compared to ZnSO_4_. Therefore, instead of Zn toxicity, it provides a sufficient amount of Zn throughout the germination period and hence improves metabolic activity and germination performance [51].

Among the various agronomic approaches to mitigate salinity stress, one of the key strategies is an external provision of plant growth regulators [52]. These plant growth regulators could potentially minimize the detrimental effects of salinity stress by regulating hormone transduction pathways and cross-talk and by manipulating the homeostatic mechanism in plants. However, the plant response differs at different growth and developmental stages [43]. Seed priming with phytochromes, as growth regulators, could be a potential approach to mitigate abiotic stresses in plants [53]. EBL is a steroidal active by-product from brassinolide biosynthesis, a member of the BRs phytochromes [54]. Seed priming with EBL in this study showed a significant improvement in fresh grain weight increase and imbibition rate under the highest salinity stress (100 mM NaCl) (Figure 1 and Figure 2). EBL at 0.4 μM concentration increased the cumulative germination percentage and germination energy while reducing the mean germination time and days to 50% emergence (Table 1). EBL treatments (T2, T3) resulted in significantly higher α-amylase activity in seeds once exposed to salinity stress (Figure 4). However, the Na^+^ content and Na^+/^K^+^ of seeds did not significantly respond to the EBL treatments (Figure 5).

On the other hand, EBL alone or in combination enhanced all investigated parameters in the current study. It can promote cell division, differentiation, and elongation, and up-regulates stress-response genes’ expression [54,55]. Under salinity-stress conditions, EBL is known for its unparalleled role in stimulating gene expression within a seed to produce the antioxidant enzymes required for the degradation of toxic substances such as reactive oxygen species (ROS) during seed germination [56]. In this study, EBL was used for seed priming considering its role in the up-regulation of stress-related genes and ability to cross-talk with other intrinsic hormones to alleviate the deleterious effects of salinity stress on seed germination. For instance, Chakma et al. [57] reported that the EBL (1.0 μM) seed priming improved germination percentage (2.5 folds), germination speed (27%), and radical development in cotton seeds under 200 mM NaCl salinity stress. Gholipoor and Roshandel [58] reported a significantly higher decline in Na^+^/K^+^ in the roots (−57.5%) in tomato seeds under 140 mM NaCl when exposed to (1.0 μM) 24-epibrassinolid.

## 4. Materials and Methods

### 4.1. Material, Seed-Priming Treatments, Preparation of Saline Soil, and Experimental Design

Grains of maize (Hybrid 310) were obtained from the Agricultural Research Center, Giza, Egypt. ZnO-NPs were purchased from Sigma-Aldrich (St. Louis, Missouri, United States), and their form was 20% (*w*/*v*) hydro-suspension with an average particle size <40 nm. EBL in powder form was purchased from ChemCruz^TM^ biochemicals (Santa Cruz Biotechnology, Huissen, Netherlands).

A sandy loam soil was collected from the Experimental Farm, King Saud University, Saudi Arabia, and were sieved through a 3 mm mesh size. Soil texture was analyzed using the Bouyoucos method [59], and it was classified as sandy loam. Cation exchange capacity (CEC) was resolved using a standard protocol as described by Richards (1954). The pH (1:2.5), EC, CEC, Na, K, and Zn of the soil used in the current study before applying the treatments were 7.85, 1.52 dS m^−1^, 6.58 cmol kg^−1^, 424 mg kg^−1^, 212 mg kg^−1^, and 16 mg kg^−1^, respectively.

Stock solutions of ZnO-NPs (100 mg L^−1^) and EBL (0.4 μM) were prepared and stored in airtight glass containers at −4 °C for further use. The grains were primed in a conical flask with the stock solutions of ZnO-NPs and EBL individually and in the following combinations: Control (only distilled water), EBL (0.2 μM), EBL (0.4 μM), ZnO-NPs (50 mg L^−1^), ZnO-NPs (100 mg L^−1^), EBL (0.2 μM) + ZnO-NPs (50 mg L^−1^), EBL (0.2 μM) + ZnO-NPs (100 mg L^−1^), EBL (0.4 μM) + ZnO-NPs (50 mg L^−1^), and EBL (0.4 μM) + ZnO-NPs (100 mg L^−1^). The seeds were incubated at 28 ±1 °C in a dark place for 24 h and then gently shaken using a reciprocal shaker (Phoenix, RS-OS20, England, UK) at 60 rpm. Three salinity levels—control (no additional NaCl), medium salinity (50 mM NaCl), and high salinity (100 mM NaCl)—were prepared and investigated in the current study.

Maize seeds primed with ZnO-NPs and EBL were sown in germination trays (50 cm × 31 cm × 6 cm) that were filled with different levels of salt-stressed soils. Primed seeds with different treatments were sown at a depth of 3 cm with a rate of one seed per cell. The seeds were weighed individually before sowing, and each cell was labeled. The germination trays were placed in a controlled chamber at +25 ± 1 °C.

The experimental design was factorial combination (27 combined treatments), in a completely randomized design (CRD), of three salt-stress treatments (0, 50, and 100 mM NaCl) and nine treatments of seed priming with ZnO-NPs, EBL, and their combinations. The number of replications for each treatment was three.

### 4.2. Measurements

#### 4.2.1. Soil Analysis before Sowing

Prior to the experiment, soil samples (in triplicate) were analyzed chemically following standard procedures [60]. Soil pH and EC were measured by preparing a soil and water suspension in a 1:2.5 *w*/*v* ratio. Samples were placed on a reciprocal shaker (Phoenix, RS-OS20, England, UK) for 1 h, followed by filtration using Whatmann 42 filter paper (GE Healthcare, Chalfont St Giles, UK). Subsequently, filtrates were analyzed using pH equipment (pH 523, WTW, Berlin, Germany) for analyzing pH, while EC was analyzed using EC equipment (EC-YSI, Model 35, Yellow Springs Instrument Co., Inc., Yellow Springs, OH, USA).

#### 4.2.2. Seed Water Uptake Pattern and Percentage Weight Increase

The amount of water uptake by the primed seeds sown in different salt-stressed soils was computed as the weight difference of grains after every 8 h for 7 d. The initial weight of each individual grain was recorded prior to sowing, and the final grain weight was recorded at 8 h intervals as follows: 8, 16, 24, 32, 40, 48, 56, 64, 72, 80, 88, 96, 104, 112, 120, 128, 136, 144, 152, and 160 h after sowing. Grains were taken from the soil, washed with distilled water, and excess water was removed from the surface using a paper towel and weighed immediately.

The percentage increase in seed weight was calculated as:

$$Percentage\;weight\;increase=\frac{Final\;weight\;–\;Initial\;weight}{Initial\;weight}\times 100$$

#### 4.2.3. Imbibition Rate

The imbibition rate was calculated as mg of water absorbed per g of dry grain weight per h using the following equation.

$$Imbibition\;rate=\frac{\left[W2-W1\;/\;W1\right]}{\left[T2-T1\right]}$$

where *W*_2_–*W*_1_ represents the grain weight difference (mg) between two consecutive readings at two different times (i.e., *T*_2_ and *T*_1_), respectively. The rate of imbibition of maize grains was expressed as the mass of water absorbed per g of seed dry weight per h of the imbibition period (mg g^−1^ h^−1^).

#### 4.2.4. α-Amylase Activity Assay

Ten grains from each of the 27 treatments were collected from the soil at 24, 48, 72, 96, and 120 h after sowing, washed with distilled water, and cleaned with tissue paper for removing extra water. A homogenized mixture was prepared by adding 100 mL of chilled distilled water in ground grains. The tubes containing the grain mixture were then dipped in a cooling bath for 10 min at +4 °C and filtered using a muslin cloth. The filtrated solution was centrifuged for 15 min at 12,298× *g*-force and +3 °C. The supernatant was carefully collected in labeled tubes which were then used as a crude extract for the α-amylase activity assay following the method described by Miller [61]. Aiming to inactivate β-amylase, the crude extract was heated for 15 min at +70 °C. A mixture of 1.0 mL of 1.0% starch solution and 1.0 mL of processed crude extraction was prepared by dissolving them in a Na-acetate buffer at pH of 5.6. Then, the mixture was incubated for 15 min at +40 °C, then 0.5 mL of 3,5-dinitrosalicylic acid was added and the mixture was boiled for 5 min in a water bath at constant temperature and then was cooled. The absorption of the reaction mixture was recorded using a spectrophotometer at 540 nm (Jenway, 6700 visible spectrophotometers, Cole-Parmer Inc., Vernon Hills, IL, USA) where maltose, reducing sugar, was used as a standard. The micromoles (μmol) of maltose produced per minute in the mixture under standard conditions were taken as a unit of α-amylase activity.

#### 4.2.5. Germination Performance

##### Cumulative Germination Percentage

The cumulative germination percentage was computed as the total germinated grains at the end of the experiment as a percentage fraction of the total number of sown grains. The grains were considered germinated once the radical reached approximately 2 mm.

$$\mathrm{Cumulative}\;\mathrm{Percentage}\;\mathrm{Germination}=\frac{\mathrm{Total}\;\mathrm{Number}\;\mathrm{of}\;\mathrm{Germinated}\;\mathrm{Grains}}{\mathrm{Total}\;\mathrm{Number}\;\mathrm{of}\;\mathrm{Tested}\;\mathrm{Grains}}\times 100$$

##### Daily Germination Response

The total number of germinated grains was recorded at ≈10:00 am daily for twelve consecutive days starting from the first grain placement in the soil. The daily germination is expressed as the daily germination percentage as a function of time.

##### Days to 50% Germination

The number of germinated grains was recorded daily consistently at 10:00–11.00 am. Days to 50% germination were counted from the first day of the sowing process to the day when half of the grains were germinated.

##### Mean Germination Time (MGT) and Germination Energy (%) at the 4th Day (%)

Mean germination time of each treatment was calculated from the daily germination data using an equation established by Dezfuli et al. [62].

MGT = ∑nD/∑n

where n is the number of newly germinated grains at day (D), D is the number of days since grain placement, and ∑n is the total number of grains germinated at the end of the experiment.

Germination energy (%) was calculated as follows:

$$Germination\;Energy=\frac{Number\;of\;germinated\;seeds\;at\;4^{\mathrm{th}}\;Day}{Total\;number\;of\;tested\;seeds}\times 100$$

#### 4.2.6. Mineral Profiling of Primed Seeds

Ten maize seeds primed with ZnO-NPs and/or EBL were randomly collected and oven-dried (+65 °C) to a constant weight, ground, and digested using sulfuric acid (H_2_SO_4_) and hydrogen peroxide (H_2_O_2_) following Wolf’s method to analyze K and Na [63]. Briefly, about 200 mg of each sample was placed in the digestion tubes followed by the addition of 2.0 mL H_2_SO_4_ (98% *w*/*w*, BDH laboratory supplies, Poole, UK). The digestion tubes were placed on the heating plate for 15 min at 220 °C until the fumes were exhausted. The successive samples were moved from the heating plate for cooling, and 1.0 mL H_2_O_2_ (33% *w*/*v*, PanReac AppliChem, Darmstadt, Germany) was added to each sample. Then, samples were placed on a heating plate at 220 °C for 30 min until the fumes were exhausted. Subsequently, 0.5 mL H_2_O_2_ was added to each sample until the solution became clear. Next, samples were cooled, filtered, and diluted with distilled water to a constant volume (i.e., 50 mL). Finally, Na and K were analyzed using a Flame photometer (Model: Corning 400, Sherwood Scientific Ltd., Cambridge, UK).

### 4.3. Statistical Analysis

The effects of salt-stressed soil as well as seed-priming treatments with ZnO-NPs, EBL, and their combinations on germination performance, α-amylase activity, and mineral profiling of primed seeds of maize were subjected to the analysis of variance (ANOVA) using PASW statistics 21.0 (IBM Inc., Chicago, IL, USA). Different means of different treatments for the same trait were compared using Tukey’s test to show the significant differences at *p* ≤ 0.05.

## 5. Conclusions

The findings of our study showed that germination characteristics such as the cumulative germination percentage, germination energy, increase in fresh grain weight, imbibition rate, metabolic activity, and ionic balance were negatively affected by salt-stress treatments (50 and 100 mM NaCl) compared to the unstressed treatment (control). However, seed priming with ZnO-NPs and EBL, both as individual treatments as well as their combinations enhanced seed germination and the metabolic and homeostatic response of maize seeds grown under salt stress compared to the control. The seed-priming combination treatments of 100 mg ZnO-NPs L^−1^ + 0.4 μM EBL and/or 100 mg ZnO-NPs L^−1^ + 0.2 μM EBL resulted in a significant enhancement for most of the germination characteristics, α-amylase activity, and ionic balance in seeds of maize grown under salt stress compared to all other individual or combined seed-priming treatments. Therefore, we recommend an extensive study with a wider range and lower interval of concentrations to develop a mathematical model between soil salinity and ZnO-NPs and EBL concentrations to recommend optimum dose as per the need of the saline soil.

## Figures and Tables

**Table 1:** Interactive effect of salinity and ZnO-NP and EBL seed-priming treatments on the cumulative germination percentage, days to 50% emergence, mean germination time, and germination energy of maize seeds.

Salinity	Treatment	Cumulative Germination (%)	Days to 50% Emergence (Days)	Mean Germination Time (Days)	Germination Energy (%)
Control (0 mM)	T1	91.33 de	4.66 e	5.08 de	31.33 bc
	T2	91.67 c	5.00 e	5.22 d	33.33 ab
	T3	91.33 de	4.67 e	4.93 de	36.67 ab
	T4	92.67 bc	4.33 e	4.93 de	39.33 a
	T5	93.67 ab	5.33 d	5.10 de	26.00 bc
	T6	93.33 ab	4.67 e	5.31 d	39.33 a
	T7	94.67 a	5.33 d	5.07 de	31.33 ab
	T8	92.67 bc	5.33 d	5.38 d	26.67 bc
	T9	94.33 a	5.33 d	5.36 d	30.67 bc
50 mM NaCl	T1	86.67 h	6.33 c	6.46 d	6.00 ef
	T2	86.67 h	6.00 c	6.08 c	14.00 d
	T3	89.67 ef	5.33 d	5.62 d	20.00 c
	T4	88.67 fg	5.33 d	5.43 d	27.33 bc
	T5	90.67 de	5.67 d	5.63 d	22.00 c
	T6	91.67 c	5.33 d	5.55 d	26.00 bc
	T7	92.67 bc	5.33 d	5.46 d	28.67 bc
	T8	92.67 bc	4.67 e	4.90 de	37.33 a
	T9	93.33 ab	5.67 d	5.61 d	29.33 bc
100 mM NaCl	T1	77.67 l	8.67 a	8.38 a	1.33 ef
	T2	81.67 k	6.67 c	6.61 c	6.67 e
	T3	83.33 j	5.67 d	5.66 d	20.67 c
	T4	81.67 k	7.33 b	6.72 c	3.33 ef
	T5	86.33 h	8.33 a	7.38 b	1.33 ef
	T6	84.67 i	7.00 b	7.00 c	4.67 ef
	T7	86.67 h	7.33 b	7.54 b	2.00 ef
	T8	85.33 i	5.67 d	5.71 d	21.33 c
	T9	86.67 h	6.33 c	6.32 d	14.00 d
SEM		0.567	0.385	0.252	3.421
*p* value		**	**	**	**

For abbreviations, please see Figure 1, SEM = standard error of means. ** indicates *p* ≤ 0.01; data followed by the same letter were not significantly different at *p* ≤ 0.05. Different letters are significantly differed at *p* ≤ 0.05.

## Data Availability

The data is contained within the manuscript.
